# 214. Prospective Evaluation of the GenMark Dx ePlex^®^ Blood Culture Identification Gram Negative Panel

**DOI:** 10.1093/ofid/ofab466.416

**Published:** 2021-12-04

**Authors:** Jeremy Meeder, Derek Moates, Hannah Pierce, Jamie Hutchinson, Pia Cumagun, Cameron White, Rachael A Lee, Todd P McCarty, Sixto M Leal

**Affiliations:** 1 University of Alabama at Birmingham, Birmingham, Alabama; 2 University of Alabama at Birmingham; Birmingham VA Medical Center, Birmingham, Alabama

## Abstract

**Background:**

The ePlex BCID Gram-Negative (GN) panel utilizes electrowetting technology to detect the most common causes of GN bacteremia (21 targets) and 6 antimicrobial resistance genes from positive blood culture bottles. Rapid detection of extended spectrum β-lactamases (ESBL; CTX-M), carbapenemases (KPC, NDM, IMP, VIM, OXA 23/48), and highly resistant bacteria such as *Stenotrophomonas maltophilia* enables early optimization of antimicrobial therapy.

**Methods:**

In this prospective study, we evaluated the performance of the BCID-GN panel compared to traditional standard of care culture and susceptibility testing with organism identification using the BioMerieux Vitek MS Matrix Assisted Laser Desorption Ionization (MALDI) Time of Flight mass spectrometry. Samples submitted for standard of care testing in Biomerieux BacT/Alert resin FA/FN blood culture bottles on the BacT/Alert VIRTUO automated blood culture system with GN bacteria on direct exam (n=108) were included.

**Results:**

All but two GN bacteria identified by MALDI were represented on the BCID-GN Panel (106/108, 98.1%) and most tests (107/108, 99.1%) yielded valid results. Discordant analyses revealed a positive percent agreement (PPA) of 102/105 (97.2%) with 3 false negatives (2 pan-susceptible *Enterobacterales*, 1 ESBL *E.coli*) and a negative percent agreement (NPA) of 105/105 (100%). Consistent with alternative resistance mechanisms, only 8/12 (66.7%) of Enterobacterales with resistance to 3^rd^ generation cephalosporins harbored the CTX-M gene. In contrast, 8/8 (100%) of isolates from samples harboring the CTX-M gene were resistant to 3^rd^ generation cephalosporins.

**Conclusion:**

Detection of 1 *S. maltophilia*, 1 *Acinetobacter baumannii* expressing OXA 23/48, and 8 Enterobacterales expressing CTX-M represent opportunities for early optimization of antimicrobial therapy in 10/108 (9.3%) of samples. The BCID-GN Panel provides rapid accurate detection of resistant gram negative bacteria enabling high quality data driven optimization of antimicrobial therapy.

**Disclosures:**

**Todd P. McCarty, MD**, **Cidara** (Grant/Research Support)**GenMark** (Grant/Research Support, Other Financial or Material Support, Honoraria for Research Presentation)**T2 Biosystems** (Consultant) **Sixto M. Leal, Jr., MD, PhD**, **Abnova** (Grant/Research Support)**AltImmune** (Grant/Research Support)**Amplyx Pharmaceuticals** (Grant/Research Support)**Astellas Pharmaceuticals** (Grant/Research Support)**CNINE Dx** (Grant/Research Support)**GenMark Diagnostics** (Grant/Research Support, Other Financial or Material Support, Honoraria- Research Presentation)**IHMA** (Grant/Research Support)**IMMY Dx** (Grant/Research Support)**JMI/Sentry** (Grant/Research Support)**mFluiDx Dx** (Grant/Research Support)**SpeeDx Dx** (Grant/Research Support)**Tetraphase Pharmaceuticals** (Grant/Research Support)

